# T cell and antibody kinetics delineate SARS-CoV-2 peptides mediating long-term immune responses in COVID-19 convalescent individuals

**DOI:** 10.1126/scitranslmed.abf7517

**Published:** 2021-03-15

**Authors:** Tatjana Bilich, Annika Nelde, Jonas S. Heitmann, Yacine Maringer, Malte Roerden, Jens Bauer, Jonas Rieth, Marcel Wacker, Sebastian Hörber, David Rachfalski, Melanie Märklin, Stefan Stevanović, Hans-Georg Rammensee, Helmut R. Salih, Juliane S. Walz

**Affiliations:** 1 Clinical Collaboration Unit Translational Immunology, German Cancer Consortium (DKTK), Department of Internal Medicine, University Hospital Tübingen, 72076 Tübingen, Germany.; 2 Institute for Cell Biology, Department of Immunology, University of Tübingen, 72076 Tübingen, Germany.; 3 Cluster of Excellence iFIT (EXC2180) “Image-Guided and Functionally Instructed Tumor Therapies”, University of Tübingen, 72076 Tübingen, Germany.; 4 Department of Hematology, Oncology, Clinical Immunology and Rheumatology, University Hospital Tübingen, 72076 Tübingen, Germany.; 5 Institute for Clinical Chemistry and Pathobiochemistry, Department for Diagnostic Laboratory Medicine, University Hospital Tübingen, 72076 Tübingen, Germany.; 6 German Cancer Consortium (DKTK) and German Cancer Research Center (DKFZ), partner site Tübingen, 72076 Tübingen, Germany.; 7 Dr. Margarete Fischer-Bosch Institute of Clinical Pharmacology and Robert Bosch Center for Tumor Diseases (RBCT), 70376 Stuttgart, Germany.

## Abstract

Long-term immunological memory to severe acute respiratory syndrome coronavirus 2 (SARS-CoV-2) is crucial for the development of population-level immunity, which is the aim of vaccination approaches. Reports on rapidly decreasing antibody titers have led to questions regarding the efficacy of humoral immunity alone. The relevance of T cell memory after coronavirus disease 2019 (COVID-19) remains unclear. Here, we investigated SARS-CoV-2 antibody and T cell responses in matched samples of COVID-19 convalescent individuals up to six months post-infection. Longitudinal analysis revealed decreasing and stable spike- and nucleocapsid-specific antibody responses, respectively. In contrast, functional T cell responses remained robust, and even increased, in both frequency and intensity. Single peptide mapping of T cell diversity over time identified open reading frame-independent, dominant T cell epitopes mediating long-term SARS-CoV-2 T cell responses. Identification of these epitopes may be fundamental for COVID-19 vaccine design.

## Teaser

Longitudinal analysis identifies SARS-CoV-2 peptide targets of durable memory T cell responses in COVID-19 convalescent individuals.

## INTRODUCTION

The severe acute respiratory syndrome coronavirus 2 (SARS-CoV-2) pandemic poses a serious threat to the world population with dramatic socioeconomic consequences. Immunity after SARS-CoV-2 infection is crucial for individual long-term protection upon virus re-exposure, but even more important to reduce transmission rates and ultimately achieve population-level immunity. Moreover, elucidation of the immunological mechanisms underlying the potential development of protective long-term immunity in the course of coronavirus disease 2019 (COVID-19) will guide the design of effective SARS-CoV-2 vaccines and treatment.

Long-term immunity is generally mediated by the adaptive immune system. Memory B and T cells persist after infection and enable more rapid and effective responses upon re-challenge with the same pathogen (*1*). However, the persistence of cellular and humoral immunological memory differs between pathogens, and experience with the other two zoonotic coronaviruses, SARS-CoV-1 and Middle East respiratory syndrome coronavirus (MERS-CoV), revealed early loss of humoral immunity (*2*, *3*). So far, data on long-term immunity to SARS-CoV-2 is limited. Available reports, up to eight months after COVID-19, are partially conflicting, but overall point toward a decrease and even loss of SARS-CoV-2-specific antibody responses (*4*–*9*) and thus raise concerns regarding long-term humoral immunity. In contrast, first reports suggest maintained cellular immunity (*10*, *11*). However, the functionality of durable SARS-CoV-2-specific T cells, as well as the exact epitopes mediating these long-term T cell responses, remain unclear. In SARS-CoV-1, T cell immunity was identified as important determinant for recovery and long-term protection (*12*–*15*), with long-lasting memory T cell responses detected in convalescent individuals even 17 years after infection (*16*). Additionally, T cell immunity also appears to play a key role in the immune response during COVID-19, with several studies reporting the presence of T cell responses in acute infection and up to eight months after convalescence (*5*, *10*, *11*, *17*–*20*). This is also supported by evidence for potential preexisting immunity mediated by cross-reactive T cells to human common cold coronaviruses (*16*, *21*–*23*). We and others recently characterized the T cell epitopes mediating these specific and cross-reactive SARS-CoV-2 T cell responses in individuals during convalescence and in unexposed individuals, providing evidence that the development of immunity requires recognition of multiple epitopes (*16*, *21*–*25*). In light of the available data on immune responses against SARS-CoV-2, persistence of SARS-CoV-2-specific T cell immunity may be crucial for long-term protection after COVID-19, which has additional consequences for vaccine development. Here, we conducted a comprehensive longitudinal analysis comparing T cell and antibody responses in SARS-CoV-2 convalescent individuals up to six months post infection. We report on the differential kinetics of cellular and humoral immunity after COVID-19 and delineate dominant peptides recognized by T cells that are essential for long-term immunity.

## RESULTS

### Longitudinal follow-up of COVID-19 convalescent donors characterized post-infectious symptoms and identified sustained SARS-CoV-2-directed T cell responses.

**Fig. 1:**
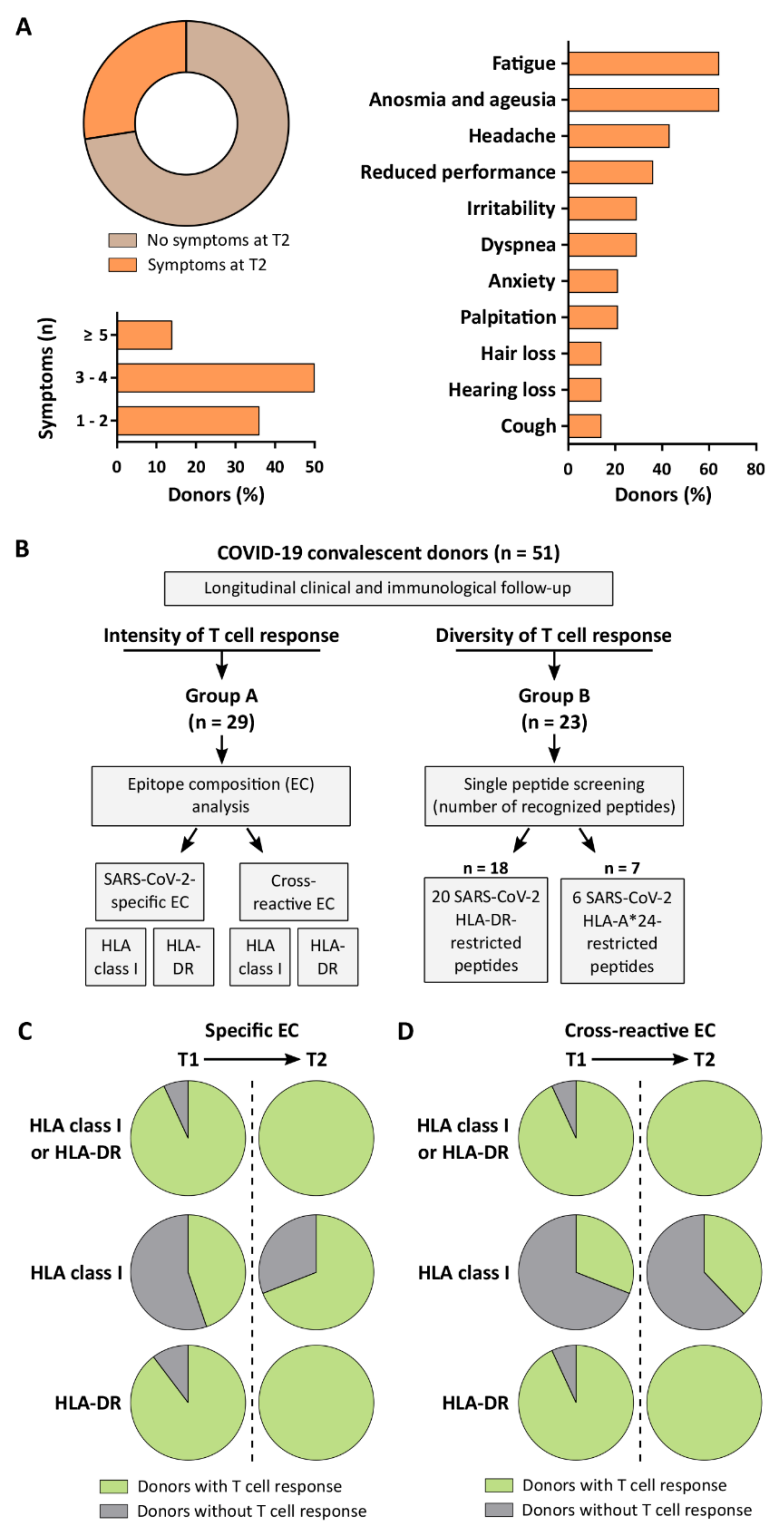
Longitudinal clinical and immunological analysis of convalescent donors after SARS-CoV-2 infection. (**A**) Prevalence, quantity and character of post-infectious symptoms in convalescent COVID-19 donors (total n = 51, symptomatic n = 14) at T2. (**B**) Schematic overview of the experimental workflow for the longitudinal analysis of immune responses in convalescent donors (n = 51). Intensity of T cell responses was assessed in group A (n = 29) using SARS-CoV-2-specific and cross-reactive epitope compositions (EC) comprising multiple HLA class I- and HLA-DR-restricted SARS-CoV-2-specific and cross-reactive T cell epitopes in ex vivo IFN-γ ELISPOT assays. Diversity of T cell responses was analyzed in group B (n = 23) by single peptide screening using 20 HLA-DR-restricted and 6 HLA-A*24-restricted SARS-CoV-2-derived peptides. (**C** and **D**) Proportion of convalescent donors with T cell responses to SARS-CoV-2-specific (C) and cross-reactive (D) EC at T1 and T2.

Clinical and immunological analysis of convalescent individuals after mild or moderate SARS-CoV-2-infection (n = 51, tables S1 and S2) was conducted 35 - 56 days (median 40 days, time point 1, T1) and 141 - 183 days (median 159 days, time point 2, T2) after positive SARS-CoV-2 polymerase chain reaction (PCR) testing (fig. S1). Persistent or newly arisen post-infectious symptoms were reported by 27% of donors at T2, with fatigue (64% of symptomatic donors) as well as anosmia and ageusia (64% of symptomatic donors) being most common (Fig. 1A, table S1). Of the donors reporting post-infectious symptoms (n = 14), no PCR retesting data was available at T2. Five of 14 (36%) donors had been retested at different time points (12 - 98 days) after their initial positive PCR test, with one donor showing a positive PCR again two weeks after the initial test (table S3). Kinetics of SARS-CoV-2-directed T cell immunity was determined longitudinally with regard to both (i) intensity (group A, n = 29) and (ii) diversity (percentage of detected peptides per donor; group B, n = 23) of CD4^+^ and CD8^+^ T cell responses (Fig. 1B). To standardize determination of changes in SARS-CoV-2 T cell response intensity over time, we employed broadly applicable human leukocyte antigens (HLA) class I- and HLA-DR-restricted SARS-CoV-2 epitope compositions (EC), as described previously (*24*). These EC comprised multiple dominant and subdominant SARS-CoV-2-specific and cross-reactive peptides, where dominant peptides were recognized by ≥ 50% of HLA class I allotype-matched donors and subdominant peptides were recognized by < 50% of donors. We evaluated T cell responses against SARS-CoV-2-specific peptides recognized exclusively in convalescent individuals after COVID-19 (specific EC) or cross-reactive peptides recognized by both convalescent donors and individuals never exposed to SARS-CoV-2 (cross-reactive EC, table S4), as described previously (*24*). The HLA class I-restricted SARS-CoV-2 T cell epitopes included in this study for either specific or cross-reactive EC were restricted to the 9 most common HLA class I allotypes covering at least one HLA allotype in more than 90% of the world’s population (*26*, *27*). The number of convalescent individuals with detectable SARS-CoV-2 T cell responses was found to increase over time, from 93% at T1 to 100% at T2, as assessed by ex vivo interferon gamma (IFN-γ) ELISPOT assays (Fig. 1, C and D). Specifically, the percentage of donors with detectable T cell responses to SARS-CoV-2-specific EC increased from 45% to 69% for HLA class I and from 90% to 100% for HLA-DR (Fig. 1C). The percentage of donors with detectable T cell responses to cross-reactive EC similarly increased (HLA class I: 31% T1 versus 38% T2; HLA-DR: 93% T1 versus 100% T2; Fig. 1D).

### The intensity of SARS-CoV-2 T cell responses was maintained for CD8^+^ T cells and increased for CD4^+^ T cells over time.

**Fig. 2:**
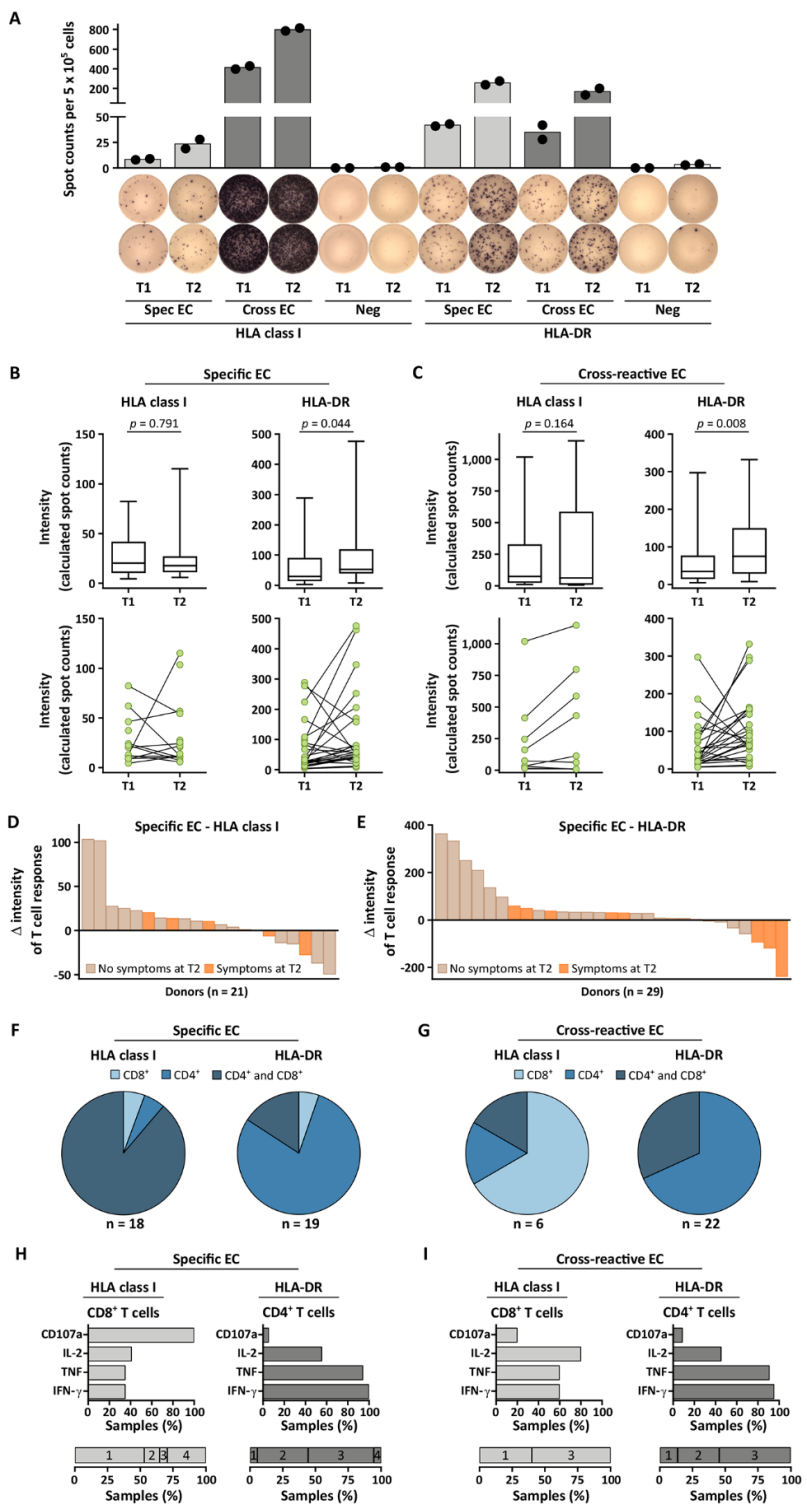
Longitudinal analysis of SARS-CoV-2 T cell response intensity in convalescent individuals. (**A**) A representative example of T cell responses to HLA class I- and HLA-DR-restricted SARS-CoV-2-specific and cross-reactive epitope compositions (EC) assessed by ex vivo IFN-γ ELISPOT assays at T1 and T2 in using cells isolated from the convalescent donor UDN121. Data are presented as scatter dot plot with bars indicating the mean spot counts of duplicates normalized to 5 × 10^5^ cells. UDN, uniform donor number; EC, epitope compositions; Spec EC, SARS-CoV-2-specific EC; Cross EC, cross-reactive EC; Neg, negative control. (**B** and **C**) Intensities of ex vivo T cell responses to SARS-CoV-2-specific (B; HLA class I-restricted, n = 21; HLA-DR-restricted, n = 29) or cross-reactive (C; HLA class I-restricted, n = 11; HLA-DR-restricted, n = 29) EC at T1 and T2. Dots represent individual donors with detectable T cell response. Data were displayed as box plots (upper row) and line plots (lower row). P values were calculated using a Wilcoxon signed rank test. (**D** and **E**) Waterfall plots show ΔT2-T1 of T cell response intensity to HLA class I-restricted (D) and HLA-DR-restricted (E) SARS-CoV-2-specific EC. Donors with post-infectious symptoms are marked in orange. (**F** and **G**) CD4^+^ and CD8^+^ T cell responses at T2 against specific (F) and cross-reactive EC (G) were evaluated by flow cytometry. (**H** and **I**) Flow cytometry-based ex vivo characterization of cytokine profiles (IFN-γ, TNF, IL-2) and degranulation marker (CD107a) for CD8^+^ and CD4^+^ T cell responses at T2 against SARS-CoV-2-specific (H) and cross-reactive EC (I). Percentage of samples with CD107a^+^, IL-2^+^, TNF^+^, and IFN-γ^+^ SARS-CoV-2 T cell responses are shown in the upper rows. The lower rows display proportion of samples revealing mono- (1), di- (2), tri- (3), or tetra-functional (4) T cell responses.

We next performed longitudinal ex vivo IFN-γ ELISPOT analysis of T cell responses from 29 individuals (group A) at T1 and T2 (Fig. 2A, fig. S2). These experiments revealed robust intensities of HLA class I-restricted SARS-CoV-2-specific and cross-reactive T cell response. In contrast, the intensities of T cell responses to HLA-DR-restricted SARS-CoV-2-specific or cross-reactive EC significantly (*p* = 0.044 and *p* = 0.008, respectively) increased over time (Fig. 2B and C). Ex vivo responses to HLA class I-restricted or HLA-DR-restricted control peptide pools derived from other viruses, including cytomegalovirus, Epstein‐Barr virus, and adenovirus peptides, showed comparable T cell response intensities at T1 and T2, with consistent intra-individual responses (n = 13, fig. S3, A to C). Accordingly, no correlation was observed in the variation of T cell responses (Δ intensity T2 - T1) to HLA-DR-restricted control peptide pools with SARS-CoV-2-specific or cross-reactive EC over time (n = 12, fig. S3, D and E). A high inter-individual heterogeneity of the intensity of longitudinal SARS-CoV-2-directed T cell responses was observed. For HLA class I-restricted SARS-CoV-2-specific EC 52% of donors showed new or ≥ 2-fold increased T cell response intensities, 24% showed stable (fold-change 0.6 - 1.9), and 24% showed ≥ 2-fold decreased or lost T cell responses at T2 (Fig. 2D). For HLA class I-restricted cross-reactive EC 45% of donors showed new or ≥ 2-fold increased T cell response intensities, 45% showed stable (fold-change 0.6 - 1.9), and 9% showed ≥ 2-fold decreased or lost T cell responses at T2 (fig. S4A). For HLA-DR, longitudinal increase of T cell response intensity in individual donors was even more pronounced, with 66% and 55% of donors with new or ≥ 2-fold increased T cell responses, 24% and 31% with stable T cell responses (fold-change 0.6 - 1.9), and 10% and 14% with ≥ 2-fold decreased or lost T cell responses to HLA-DR-restricted SARS-CoV-2-specific and cross-reactive EC at T2, respectively (Fig. 2E, fig. S4B). Interestingly, each of the three donors showing the most pronounced decrease of T cell responses to the HLA-DR-restricted SARS-CoV-2-specific EC still suffered from post-infectious symptoms (Fig. 2E). Characterization of long-term SARS-CoV-2-directed T cells at T2 using ex vivo flow cytometry-based assessment of surface markers and intracellular cytokine staining (ICS) revealed that T cell responses to HLA class I-restricted cross-reactive EC were predominantly mediated by CD8^+^ T cells, whereas T cell responses to HLA-DR-restricted SARS-CoV-2-specific and cross-reactive EC were mainly mediated by CD4^+^ T cells (Fig. 2, F and G, fig. S5). The vast majority of T cell responses to HLA class I-restricted SARS-CoV-2-specific EC were mediated by both CD8^+^ and CD4^+^ T cells (Fig. 2F), which is an often described phenomenon especially in viral disease (*28*, *29*). CD8^+^ T cells targeting HLA class I-restricted SARS-CoV-2-specific EC were mainly positive for CD107a, whereas CD4^+^ and CD8^+^ T cells targeting HLA-DR-restricted SARS-CoV-2-specific EC displayed positivity for several of the markers interleukin (IL)-2, tumor necrosis factor (TNF), IFN-γ, and CD107a (Fig. 2H). For HLA-DR- and HLA class I-restricted cross-reactive EC multifunctional T cell responses (IL-2, TNF, IFN-γ, CD107a) could also be observed (Fig. 2I). Longitudinal ICS and surface marker analyses of T cell responses at T1 and T2 further validated robust HLA class I-restricted (fig. S6) and HLA-DR-restricted (fig. S7) SARS-CoV-2-specific and cross-reactive T cell responses over time (fig. S8).

### Longitudinal SARS-CoV-2 antibody responses showed differential dynamics over time and correlation to post-infectious clinical status.

**Fig. 3:**
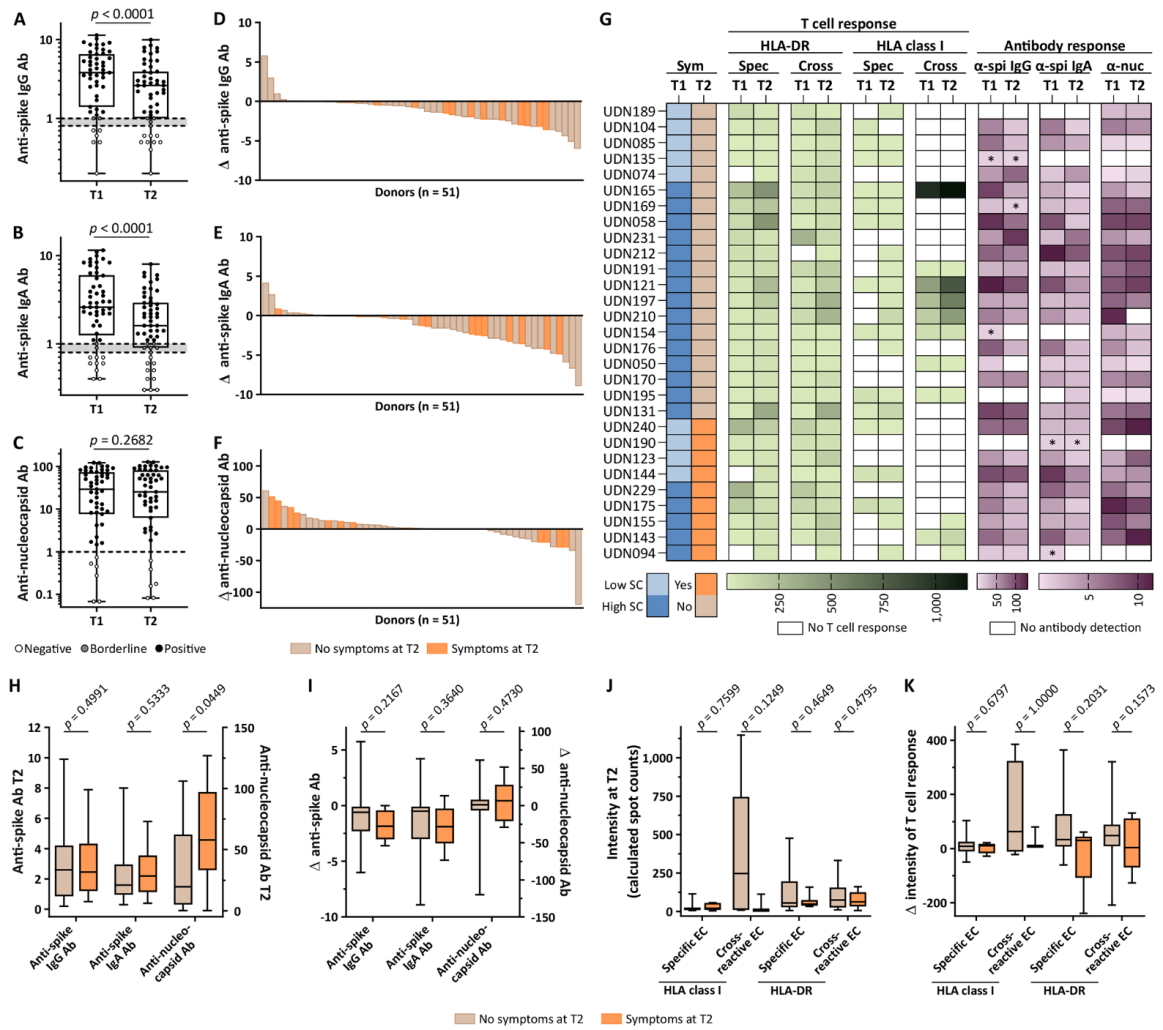
Dynamics of SARS-CoV-2 antibodies in relation to T cell responses and post-infectious clinical status. (**A** to **C**) Antibody responses in convalescent donors (n = 51) at T1 and T2 for anti-S1 IgG (A) and IgA (B) or anti-nucleocapsid (C) antibodies. Donors with negative or borderline responses are marked in white or grey, respectively. Box plots are shown. P values were calculated using a Wilcoxon signed rank test. Ab, antibody. (**D** to **F**) Waterfall plots show change of anti-S1 IgG (D) and IgA (E) ratios or anti-nucleocapsid antibody titers (F) from T1 to T2 (Δ = T2-T1). Donors with post-infectious symptoms are marked in orange. (**G**) Heatmap of COVID-19 symptom scores (SC) and post-infectious symptoms, intensities of T cell responses to different EC (color gradient green) and antibody responses (color gradient purple) at T1 and T2 in individual donors (group A, n = 29). UDN, uniform donor number; Sym, symptoms; Spec, SARS-CoV-2-specific EC; Cross, cross-reactive EC; α-nuc, anti-nucleocapsid; α-spi, anti-spike; *, donors with borderline response. (**H**) Anti-S1 IgG and IgA ratios and anti-nucleocapsid titers at T2. (**I**) ΔT2-T1 of respective antibody responses. (**J**) Intensity of T cell responses at T2 and (**K**) ΔT2-T1 of intensity to SARS-CoV-2-specific EC restricted to HLA class I (n = 21) or HLA-DR (n = 29) and cross-reactive EC restricted to HLA class I (n = 11) or HLA-DR (n = 29). Data presented as box plots. P values were calculated using Mann-Whitney U tests.

Two independent assays were employed to longitudinally assess SARS-CoV-2 antibody responses in convalescent donors (n = 51) at T1 and T2 to determine (i) ratios of IgG and IgA antibodies targeting the S1 domain of the spike protein, including the immunologically relevant receptor binding domain (RBD; Fig. 3, A and B, fig. S9, A and B) as well as (ii) anti-nucleocapsid antibody titers (Fig. 3C, fig. S9C). Both anti-S1 IgG and IgA response significantly (*p* < 0.0001) decreased over time (median 3.8 versus 2.6 and 2.6 versus 1.6, respectively), whereas anti-nucleocapsid antibody titers remained stable from T1 to T2 (median 29 versus 25). Loss or ≥ 2-fold decrease of anti-S1 IgG and IgA was observed in 31% and 44% of donors, respectively (Fig. 3, D and E), whereas loss or ≥ 2-fold decrease of anti-nucleocapsid antibody titers was documented in only 13% of donors (Fig. 3F). Among those still suffering from post-infectious symptoms at T2, 36% (5/14) and 50% (7/14) presented with ≥ 2-fold decrease or loss of anti-S1 IgG and IgA, respectively, whereas none showed a comparable decrease in anti-nucleocapsid antibody titers (Fig. 3, D to F). Anti-S1 IgG antibody responses moderately correlated with the intensity of T cell responses to HLA-DR-restricted SARS-CoV-2-specific or cross-reactive EC, as well as HLA class I-restricted cross-reactive EC at T2 (fig. S10). Longitudinal T cell and antibody responses, as well as symptoms during and after COVID-19, varied among the donors (Fig. 3G). Neither the intensity of SARS-CoV-2-specific nor that of cross-reactive T cell responses to HLA class I- or HLA-DR-restricted EC at T2 correlated with demographics (table S5). High anti-nucleocapsid antibody titers at T2 were associated with a higher prevalence of post-infectious symptoms (Fig. 3, H and I). In contrast, neither intensity nor longitudinal kinetics of SARS-CoV-2 T cell responses were associated with post-infectious symptoms (Fig. 3, J and K).

### Diversity of SARS-CoV-2 T cell immunity identifies peptides mediating long-term T cell responses.

**Fig. 4:**
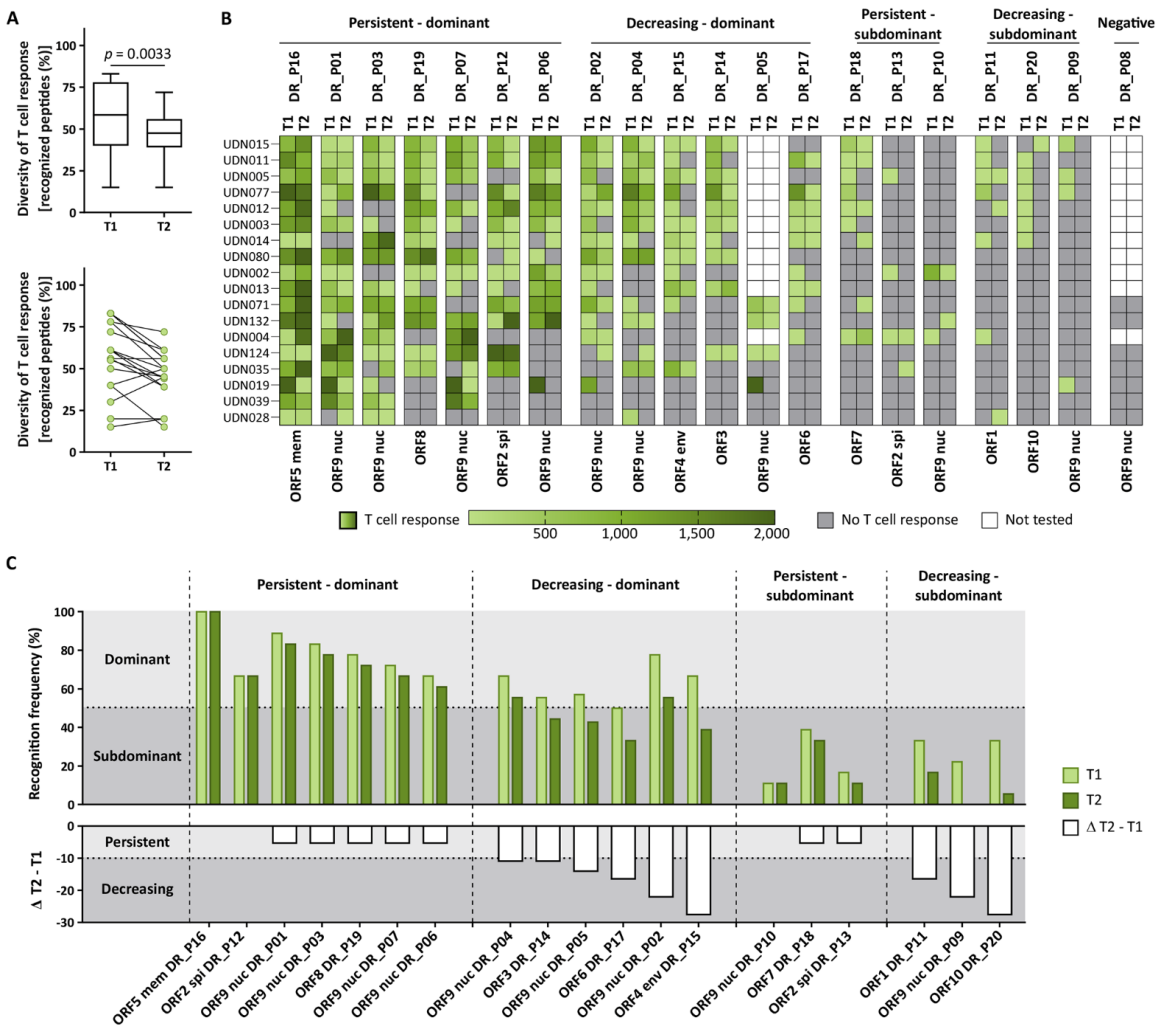
Longitudinal assessment of HLA-DR-directed T cell response diversity in convalescent donors. (**A**) Diversity of T cell responses, which refer to the percentage of recognized HLA-DR-restricted peptides per donor (n = 18; group B), at T1 and T2 as analyzed by IFN-γ ELISPOT assays after 12-day in vitro pre-stimulation. Dots represent individual donors. Data were displayed as box plots (upper row) and line plots (lower row). P value was calculated using a Wilcoxon signed rank test. (**B**) Heatmap indicating positive (green) and negative (grey) T cell responses, as well as the intensities of T cell responses (color gradient green) to 20 HLA-DR-restricted SARS-CoV-2-derived peptides (DR_P01 - DR_P20) in individual donors (n = 18) at T1 and T2. (**C**) Recognition frequencies of peptides at T1 and T2 (top) and their variation over time (bottom, ΔT2-T1) grouped into dominant and subdominant peptides capable (persistent) or incapable (decreasing) of mediating persisting T cell response over time. ORF, open reading frame; nuc, nucleocapsid; spi, spike protein; env, envelope protein; mem, membrane protein.

In various viral diseases, including COVID-19, diversity of T cell responses, which means the recognition of multiple T cell epitopes, has been implicated as a prerequisite for effective immunity (*24*, *30*). We longitudinally analyzed the diversity of SARS-CoV-2 T cell responses by single peptide mapping using dominant and subdominant promiscuous HLA-DR- (binding to several HLA-DR allotypes, n = 20) and HLA-A*24-restricted (n = 6) SARS-CoV-2-derived peptides, as described previously (*24*). To enable detection of low-frequent peptide-specific T cell populations, we used an in vitro 12-day pre-stimulation to expand SARS-CoV-2-specific T cells. Longitudinal diversity of HLA-DR- and HLA-A*24-directed T cell responses decreased across all donors and peptides over time (median T cell recognition per donor 59% and 50% at T1, 48% and 17% at T2, respectively; Fig. 4A, fig. S11). The decrease in HLA-DR-directed T cell diversity was confirmed in subgroup analyses for specific and cross-reactive peptides (fig. S12A) and dominant and subdominant peptides (fig. S12B). The decrease in diversity was also confirmed for peptides derived from structural or non-structural (fig. S12C) and nucleocapsid versus non-nucleocapsid viral open reading frames (ORF, fig. S12D). T cell response intensity after in vitro 12-day pre-stimulation showed high inter-individual and high inter-peptide heterogeneity (fig. S13). For 88% of SARS-CoV-2 HLA-DR- and HLA-A*24-restricted peptides, expansion ability of SARS-CoV-2 T cells did not differ significantly between T1 and T2 (fig. S13).

Further, donor- and peptide-specific assessment identified a subset of peptides derived from different ORFs that sustain a persistent T cell response (10/20 HLA-DR-restricted peptides, Fig. 4, B and C, table S6; 2/6 HLA-A*24-restricted peptides, fig. S11, B and C, table S7). In particular, the seven dominant HLA-DR-restricted peptides that mediated a persistent T cell response in convalescent individuals appeared to be essential for long-term T cell immunity to SARS-CoV-2 and may thus enable the development of effective vaccination approaches.

## DISCUSSION

The SARS-CoV-2 pandemic results in dramatic worldwide consequences for health care, the economy, and daily life. To enable the development of therapeutic and prophylactic interventions for COVID-19, elucidation of the mechanisms underlying SARS-CoV-2-directed immune responses is of the utmost importance. This holds particularly true for the assessment of immunological memory, which requires a detailed longitudinal analysis of cellular and humoral immune responses. Accumulated evidence obtained from patients and convalescents regarding frequency, intensity, and diversity of T cell responses and their correlation with SARS-CoV-2 antibody titers, as well as clinical characteristics, point to a central role of T cell immunity in COVID-19 (*5*, *16*, *18*, *19*, *24*, *31*). Here, we present a comprehensive longitudinal analysis of donors with SARS-CoV-2 infection over a six-month follow-up period comprising SARS-CoV-2 antibody and T cell responses as well as clinical symptoms of acute and post-COVID-19 disease.

We observed robust and increasing intensity of SARS-CoV-2 T cell responses targeting HLA class I- and HLA-DR-restricted peptides over time. This observation is in line and extends the findings of a report on SARS-CoV-2 immune memory up to three months post-infection as well as data obtained from individuals following SARS-CoV-1 infection (*3*, *5*, *16*). Consistent with previous work by our group (*24*), no difference in frequency nor in intensity was observed between T cell responses to cross-reactive or specific EC at the six-month follow-up time point. This suggests that there are no differences between SARS-CoV-2-derived peptides with and without similarities to human common cold coronaviruses in terms of mediating acute and long-term immune responses in COVID-19. The recognition of the SARS-CoV-2-derived HLA-DR-presented peptides not only by CD4^+^ but, to a lesser degree, also by CD8^+^ T cells is due to several embedded SARS-CoV-2 HLA class I-presented peptides within the HLA-DR-binding sequences. HLA-DR-restricted epitopes with embedded HLA class I-binding peptides, to induce both CD4^+^ and CD8^+^ T cell responses, are widely used for anti-cancer as well as anti-viral immunotherapy (*32*, *33*). On the other hand, we could show that the SARS-CoV-2 HLA class I-restricted EC could also be recognized by CD4^+^ T cells, which is an often described phenomenon, especially in viral diseases (*28*, *29*), as both HLA class I and class II molecules could bind to primary and secondary peptide anchor motifs covering the central 9 - 10 amino acids.

Previous data on acute and chronic viral infection (*34*–*36*), including SARS-CoV-1 and MERS-CoV, as well as several studies analyzing SARS-CoV-2 T cell responses during or early after COVID-19, have shown that CD4^+^ T cells play a central role in cellular immunity to SARS-CoV-2. This is reflected by a higher frequency of convalescent donors with detectable SARS-CoV-2-specific CD4^+^ T cells, as well as an increased T cell response intensity and broader cytokine profile of CD4^+^ T cells compared to CD8^+^ T cells (*24*, *37*, *38*). This important role of CD4^+^ T cells in the control of primary SARS-CoV-2 infection might also continue for the development of long-term immunity, reflected by the here reported trend to increased SARS-CoV-2-specific and cross-reactive HLA-DR-restricted T cell responses at six months post infection. This is in line with several studies showing a correlation of the frequency of epitope-specific naïve CD4^+^ T cells with memory repertoire for different pathogens (*39*, *40*). T cell responses to viral infections are considered to occur rapidly and peak about one week after acute infection before they decline (*34*, *41*). In contrast, data from the two previous coronavirus pandemics/epidemics mediated by SARS-CoV-1 and MERS-CoV revealed different kinetics of T cell responses. Strong ex vivo SARS-CoV-1-specific T cell responses were detected in convalescent donors even seven to eight months after infection (*36*) and memory T cell responses could be detected after in vitro expansion up to 17 years after infection (*16*). This is in line with first reports on persisting SARS-CoV-2 T cell responses after three months (*5*) as well as our data in this six-month follow-up analysis. Several studies have reported prolonged SARS-CoV-2 antigen persistence that might represent a continuous trigger for T cell responses (*42*–*46*). Furthermore, a prolonged “inflammatory state” with persistent activation of different components of the immune system has been described after COVID-19 (*47*, *48*). This might trigger an ongoing stimulation of T cells by different components of the immune system, which is of particular importance for the development and persistence of memory CD4^+^ T cells (*49*–*53*). The interaction of B cells and T cells might play an essential role and could explain the increase in SARS-CoV-2-specific and cross-reactive CD4^+^ T cell responses (*50*, *54*). This is supported by data from Dan and colleagues, showing a trend toward an increase of SARS-CoV-2-specific CD4^+^ follicular helper T cells (*11*), a specialized subset of CD4^+^ T cells required for B cell help (*55*), six months after infection.

In contrast to the kinetics of the T cell response, both IgG and IgA antibody responses to the S1 domain of the spike protein declined during the six-month follow-up. In line with several reports on decreasing antibody titers after SARS-CoV-2 infection (*5*–*8*, *34*, *35*), this finding raises concerns that humoral immunity against SARS-CoV-2 may not provide long-term protection. It needs to be taken into consideration that the protective efficacy of the antibodies analyzed in our study remains unclear, even if RBD antibody titers reportedly correspond to virus-neutralizing activity (*56*). Epidemiologic studies employing neutralizing assays in large cohorts are required to thoroughly unravel the relevance of long-term SARS-CoV-2 humoral immunity. Nevertheless, our finding that anti-S1 antibody responses decrease over time, whereas anti-nucleocapsid antibody titers persist, is important in the context of vaccine development, as several ongoing approaches are focusing on the induction of immune responses to the RBD of the spike protein (*57*, *58*).

As more than 50 million people have recovered from COVID-19, increasing evidence for the prevalence and nature of post-infectious symptoms and secondary damages is arising (*59*–*65*). There are only limited datasets (*66*–*68*) available reporting on the prevalence and nature of post-infectious symptoms after COVID-19, which was just recently claimed in a comprehensive review (*69*). This is especially true for mild COVID-19. Several large cohort studies are ongoing. Our work provides insight into post-infectious symptoms in a cohort of individuals with a mild to moderate course of COVID-19, showing post-infectious symptoms in 27% of the donors, which is in line with a very recent publication in an out-patient setting (*70*). The pathomechanism underlying persistence or development of these symptoms after SARS-CoV-2-infection is matter of investigation (*62*, *71*). Microangiopathic cerebral lesions (*72*), effects directly mediated by the virus, such as virus persistence (*42*, *43*, *45*, *46*), and immune-mediated inflammatory syndromes (*73*, *74*) are proposed to play a role. Previous studies reported on the correlation of high antibody titers with more severe course of acute COVID-19 (*6*, *24*). Here we could show that high nucleocapsid antibody titers at six-month follow-up also associate with an increased prevalence of post-infectious symptoms. No correlation of post-infectious symptoms with intensity or longitudinal dynamics of anti-SARS-CoV-2 T cell responses was observed. Together with recent data providing evidence that the intensity of T cell responses does not correlate with acute COVID-19 severity (*21*, *24*), this finding is of high relevance for the design of vaccines, as it provides evidence that disease-aggravating effects might not hamper the development of vaccination approaches aiming to induce SARS-CoV-2-specific T cell responses. Future studies are needed to validate these findings in larger cohorts and to delineate potential immune- or antibody-mediated mechanisms of post-infectious symptoms.

Previous work on viral diseases including SARS-CoV-2 implicates diversity of T cell responses, or the recognition of multiple T cell epitopes, as important prerequisite for effective immunity (*24*, *30*). Identification of respective T cell epitopes that induce potent and long-lasting SARS-CoV-2-specific responses is fundamental for both detection of immunological memory and vaccine design. Our longitudinal analysis of T cell response diversity using a single peptide-based approach allowed for discrimination of HLA-DR- and -A*24-restricted peptides capable or incapable to induce persisting SARS-CoV-2-specific or cross-reactive T cell responses. This enabled the characterization of a first set of ORF-independent, dominant T cell epitopes that may govern long-term SARS-CoV-2 T cell immunity. To expand this panel of highly promising candidate peptides for vaccine design, future studies are warranted that broadly evaluate long-term SARS-CoV-2 T cell responses to T cell epitopes of further HLA class I allotypes. In contrast, the promiscuous HLA-DR-binding peptides identified to mediate long-term T cell responses in up to 100% of donors independently of their HLA-DR allotype represent broadly applicable candidates for vaccine design. The phenomenon that dominant HLA-DR-restricted epitopes are associated with promiscuous HLA class II binding, defined as the capacity to bind multiple HLA allelic variants, is well described for other infectious diseases, including tuberculosis and malaria (*75*–*77*), and was just recently also proven for a panel of dominant SARS-CoV-2 HLA-DR-restricted T cell epitopes (*78*). The promiscuous HLA-DR-presented SARS-CoV-2-derived peptides defined here thus already constitute the basis of a multi-peptide vaccine for induction of T cell immunity to SARS-CoV-2 to combat COVID-19, which is currently evaluated in a first-in-human clinical trial (NCT04546841).

Caveats of this study include the limited follow-up time, small sample size, and the focus on non-severe courses of COVID-19. Sample size and follow-up time were limited by expedience. The focus on non-severe cases of COVID-19 also represents a strength, as long-term persistence of SARS-CoV-2 immune responses in this large group of COVID-19 convalescent donors is of utmost importance for the development of population-level immunity. Completion of this data requires large cohort studies, including longitudinal sampling of donors with severe COVID-19 over a longer period of time to (i) delineate the mechanistic basis of SARS-CoV-2 long-term immunity and (ii) confirm presence of long-term protective T cell immunity to SARS-CoV-2 based on monitoring of convalescent individuals upon virus rechallenge. Additionally, in the light of further upcoming data on longitudinal SARS-CoV-2 immune responses (*11*), future analyses are required to characterize and differentiate T cell subsets and their respective functionality state that mediated long-term T cell immunity and define the T cell epitopes, epitope compositions or peptide megapools that optimally enable their detection.

In conclusion, our data provides important insights into the differential dynamics of SARS-CoV-2-directed antibody and T cell immune responses over time, their correlation to post-COVID-19 illness, and the identity of SARS-CoV-2 peptide targets for durable memory T cell responses after COVID-19. Together, these data have broad implications for both detection and understanding of immunological memory as well as vaccine design.

## MATERIALS AND METHODS

### Study design

We performed a single-center study carried out at the Clinical Collaboration Unit Translational Immunology, University Hospital Tübingen, Germany analyzing convalescent adults after SARS-CoV-2 infection (n = 51). The study aimed to longitudinally determine the kinetics of the SARS-CoV-2-directed immune response in terms of intensity and diversity of T cell response, as well as antibody response. Blood and serum samples were collected and a questionnaire-based assessment of donor characteristics and disease symptoms during and after COVID-19 from SARS-CoV-2 convalescent donors was obtained between 4/2020 and 5/2020 (time point T1) and in 08/2020 (time point T2). Informed consent was obtained in accordance with the Declaration of Helsinki protocol. The study was approved by and performed according to the guidelines of the local ethics committees (179/2020/BO2).

The donors were analyzed in two groups for (i) intensity of CD4^+^ and CD8^+^ T cell responses (group A, n = 29) and for (ii) diversity of T cell responses (the number of detected peptides per donor; group B, n = 23). Donors analyzed in each group were identical at T1 and T2 and were assigned to the groups according to time of sample acquisition and available sample cell number as well as HLA allotype restriction (for HLA-A*24 T cell diversity assessment). One donor was analyzed in both group A and B (table S2). All analyzed donors and data were shown. Outliers were not excluded from the analyses.

SARS-CoV-2 infection was confirmed by PCR test after nasopharyngeal swab. Donor recruitment was performed by online- and paper-based advertising (homepage, flyer). Sample collection was performed in a longitudinal manner approximately 35 - 56 days (T1) and 141 - 183 days (T2) after positive PCR. Samples were processed in the Department of Immunology located at the same hospital site. Peripheral blood mononuclear cells (PBMCs) were isolated by density gradient centrifugation and used directly or stored at -80°C until further use. Serum was separated by centrifugation for 10 min and the supernatant was stored at -80°C. Flow cytometry- and T cell-based experiments were conducted at the Department of Immunology, aliquots were also shipped at -20°C to the Department of Clinical Chemistry and Pathobiochemistry for antibody analysis.

HLA typing was carried out by Immatics Biotechnology GmbH and the Department of Hematology and Oncology at the University Hospital Tübingen. Symptom score (SC) to assess severity of COVID-19 was determined by combining objective (fever ≥ 38.0°C) and subjective disease symptoms (no/mild/moderate versus severe, reported by questionnaire) of individual donors. Donors with severe disease symptoms or fever were classified as “high SC”, all others as “low SC”. In addition, subjective post-infectious complaints and symptoms were assessed at the follow-up sample acquisition (T2). Detailed donor characteristics as well as information on allocation of the donors to the experimental groups are provided in tables S1 and S2.

### SARS-CoV-2 peptides

Synthetic peptides were provided by EMC Microcollections GmbH and INTAVIS Peptide Services GmbH & Co. KG. The HLA class I- and HLA-DR-restricted peptides as well as the applied EC were characterized in detail in a previous work (*24*) analyzing T cell responses in convalescent individuals after COVID-19 as well as in healthy donors never exposed to the virus. T cell epitopes were defined as dominant if immune responses against these peptides were detected in ≥ 50% of convalescent donors. For HLA class I-restricted peptides, T cell responses and definition of dominance were only assessed in HLA-matched donors. The SARS-CoV-2 HLA class I-restricted peptides used in this study were predicted to bind to a specific HLA allotype and validated in COVID-19 convalescent donors with this respective HLA allotype, whereas promiscuous SARS-CoV-2-derived HLA-DR-restricted peptides binding to several HLA-DR allotypes were selected and validated independent of the HLA-DR allotype.

The term “cross-reactive peptide” is used for SARS-CoV-2 peptides not only eliciting T cell responses in donors after SARS-CoV-2 infection, but also in unexposed individuals. To delineate any differences in SARS-CoV-2 T cell responses to specific or cross-reactive peptides in the long-term follow-up, we included the cross-reactive HLA class I- and HLA-DR-restricted EC in this longitudinal analysis.

For the determination of ex vivo intensity of SARS-CoV-2 T cell responses at T1 and T2, standardized and previously validated HLA class I- and HLA-DR-restricted SARS-CoV-2-specific and cross-reactive EC were applied (table S4) (*24**, **78*). As the determination of T cell response diversity requires the analysis of multiple peptides, we used HLA allotypes with several validated SARS-CoV-2 peptides including HLA-DR (20 peptides with multiple HLA-DR restrictions) and HLA-A*24 (6 peptides).

### IFN-γ enzyme-linked immunospot (ELISPOT) assays

HLA class I- and HLA-DR-restricted SARS-CoV-2-specific and cross-reactive EC and single peptides were used for longitudinal analysis of T cell response intensity and diversity, respectively, comprising multiple dominant (recognized by ≥ 50% of donors) and subdominant (recognized by < 50% of donors) SARS-CoV-2-specific peptides recognized exclusively in COVID-19 convalescent donors or cross-reactive peptides recognized by both convalescent donors and individuals never exposed to SARS-CoV-2 (table S4), as described previously (*21*).

For the longitudinal analysis of T cell response intensity, freshly isolated or thawed PBMCs were pulsed with either SARS-CoV-2-specific or cross-reactive EC (HLA class I- or HLA-DR-restricted) and analyzed directly ex vivo by IFN-γ ELISPOT assay in duplicates. Ex vivo responses to a control peptide pool, including peptides derived from cytomegalovirus, Epstein-Barr virus, and adenovirus (table S8), served as controls. T cell diversity (percentage of recognized peptides) was analyzed following 12-day in vitro pre-stimulation prior to single peptide analysis (HLA-DR- or HLA-A*24-restricted peptides) by IFN-γ ELISPOT assay. For pre-stimulation, PBMCs were pulsed with HLA-A*24- (1 μg/mL) or HLA-DR-restricted peptide pools (5 μg/mL) and cultured for 12 days under addition of IL-2 (20 U/mL, Novartis) on days 2, 5, and 7. Expanded PBMCs were analyzed by single peptide readout ELISPOT in duplicates. Up to 8 × 10^5^ cells per well were co-incubated in 96-well plates with 1 μg/mL of HLA class I-restricted or 2.5 μg/mL of HLA-DR-restricted peptide pools directly ex vivo (for EC) or of single peptides following the 12-day T cell expansion. 96-well plates were coated with 2 μg/mL anti-IFN-γ antibody (clone 1-D1K, MabTech, Cat# 3420-3-250, RRID: AB_907283). After a 24 hour incubation, spots were revealed with 0.3 μg/mL anti-IFN-γ biotinylated detection antibody (clone 7-B6-1, MabTech, Cat# 3420-6-250, RRID: AB_907273), ExtrAvidin-Alkaline Phosphatase (1:1,000 dilution, Sigma-Aldrich), and BCIP/NBT (5-bromo-4-chloro-3-indolyl-phosphate/nitro-blue tetrazolium chloride, Sigma-Aldrich). Phytohemagglutinin (PHA, Sigma-Aldrich) served as positive control. Irrelevant HLA-matched control peptides (HLA-A*24, AYVHMVTHF, BI1_HUMAN_45-53_ and HLA-DR, ETVITVDTKAAGKGK, FLNA_HUMAN_1669−1683_) or, in case of HLA class I-restricted EC, 10% dimethyl sulfoxide (DMSO) in double-distilled water (ddH_2_O) served as negative control. Spots were counted using an ImmunoSpot S5 analyzer (CTL) and T cell responses were considered positive when the mean spot count was ≥ 3-fold higher than the mean spot count of the negative control. The intensity of T cell responses is depicted as calculated spot counts, which represent the mean spot count of duplicates normalized to 5 × 10^5^ cells minus the normalized mean spot count of the respective negative control (as in Fig. 2 B and C, Fig. 3, G, J and K, fig. S3, fig. S8, C and D, fig. S10, fig. S13). The recognition frequency of T cell responses within a donor group indicates the relative number of donors which can recognize the respective EC or peptide (positive donors/tested donors) (as in Fig. 1, C and D, Fig. 4C, fig. S11C, tables S6 and S7). The diversity of T cell responses for single donors represents the number of recognized SARS-CoV-2-derived peptides (positive peptides/tested peptides) (as in Fig. 4A, fig. S11A, fig. S12).

### Intracellular cytokine and cell surface marker staining

Peptide-specific T cells were characterized by intracellular cytokine and cell surface marker staining. PBMCs were incubated with 10 μg/mL per peptide of EC or negative control peptide, 10 μg/mL Brefeldin A (Sigma-Aldrich), and a 1:500 dilution of GolgiStop (BD Biosciences) for 12 - 14 hours. Staining was performed using Cytofix/Cytoperm solution (BD Biosciences), APC/Cy7 anti-human CD4 (1:100 dilution, BioLegend, Cat# 300518, RRID: AB_314086), PE/Cy7 anti-human CD8 (1:400 dilution, Beckman Coulter, Cat# 737661, RRID: AB_1575980), Pacific Blue anti-human TNF (1:120 dilution, BioLegend, Cat# 502920, RRID: AB_528965), FITC anti-human CD107a (1:100 dilution, BioLegend, Cat# 328606, RRID: AB_1186036), and PE anti-human IFN-γ monoclonal antibodies (1:200 dilution, BioLegend, Cat# 506507, RRID: AB_315440). T cells exposed to phorbol myristate acetate (PMA, 5 μg/mL, Sigma-Aldrich) and ionomycin (1 μM, Sigma-Aldrich) served as positive controls. Viable cells were determined using Aqua live/dead (1:400 dilution, Invitrogen). All samples were analyzed on a FACS Canto II cytometer (BD Biosciences) and evaluated using FlowJo software version 10.0.8 (BD Biosciences). The gating strategy applied for the analyses of flow cytometry-acquired data is provided in fig. S14.

### SARS-CoV-2 IgG and IgA ELISA (EUROIMMUN)

The 96-well SARS-CoV-2 IgG and IgA ELISA assay (EUROIMMUN, 2606A_A_DE_C03, as constituted on 22 April 2020) was performed on an automated BEP 2000 Advance system (Siemens Healthcare Diagnostics GmbH) according to the manufacturer’s instructions. The ELISA assay detects anti-SARS-CoV-2 IgG and IgA directed against the S1 domain of the viral spike protein (including the immunologically relevant RBD) and relies on an assay-specific calibrator to report a ratio of specimen absorbance to calibrator absorbance. The final interpretation of positivity is determined by ratio above a threshold value given by the manufacturer: positive (ratio ≥ 1.1), borderline (ratio 0.8 - 1.0), or negative (ratio < 0.8). Quality control was performed following the manufacturer’s instructions on each day of testing.

### Elecsys anti-SARS-CoV-2 immunoassay

The Elecsys anti-SARS-CoV-2 assay is an ECLIA (electrogenerated chemiluminescence immunoassay) designed by Roche Diagnostics GmbH and was used according to the manufacturer’s instructions (V1.0, as constituted in May 2020). It is intended for the detection of high-affinity antibodies (including IgG) directed against the nucleocapsid protein of SARS-CoV-2 in human serum. Readout was performed on the Cobas e411 analyzer (Roche Diagnostics). Negative results were defined by a cut-off index of < 1.0. Quality control was performed following the manufacturer’s instructions on each day of testing.

### Software and statistical analysis

Flow cytometric data was analyzed using FlowJo 10.0.8 (BD Biosciences). Data are displayed as mean with standard deviation (for n ≥ 3), scatter dot plot with mean, box plot as median with 25th or 75th percentiles, min/max whiskers, or violin plots with median and quartiles. Descriptions of the statistical tests that were used for evaluation of the experiments are provided within the respective figure legends. Continuous data were tested for distribution, and D’Agostino’s K^2^ test was used as a normality test. Individual groups were tested by use of paired Wilcoxon or unpaired Mann-Whitney U tests and tests were two-sided. Spearman’s rho (ρ) was calculated for correlation between continuous data. Missing data were included in tables and in descriptive analysis. Graphs were plotted using GraphPad Prism 8.4.3. Statistical analyses were conducted using GraphPad Prism 9.0.2 and JMP Pro (SAS Institute Inc., version 14.2) software. P values of < 0.05 were considered statistically significant.

## Supplementary Materials

stm.sciencemag.org/cgi/content/full/scitranslmed.abf7517/DC1 Fig. S1. Schematic overview and timeline of the experimental setting for the longitudinal analysis of convalescent donors after SARS-CoV-2 infection Fig. S2. IFN-γ ELISPOT assays of longitudinal SARS-CoV-2 T cell responses in convalescent individuals Fig. S3. Control ex vivo IFN-γ ELISPOT assay for validation of T cell response intensities between T1 and T2 Fig. S4. Kinetics of T cell response intensity to cross-reactive epitope compositions in individual convalescent donors Fig. S5. Flow cytometry-based characterization of long-term SARS-CoV-2-specific T cell responses Fig. S6. Flow cytometry-based characterization of CD8^+^ T cell responses at T1 and T2 Fig. S7. Flow cytometry-based characterization of CD4^+^ T cell responses at T1 and T2 Fig. S8. Longitudinal flow cytometry-based analysis of SARS-CoV-2 T cell response Fig. S9. Longitudinal assessment of SARS-CoV-2-directed antibody responses Fig. S10. Correlation of SARS-CoV-2 antibody response with intensity of T cell responses at T2 Fig. S11. Diversity of T cell responses to HLA-A*24-restricted peptides in convalescent donors over time Fig. S12. Longitudinal assessment of SARS-CoV-2 T cell response diversity in different subgroups of HLA-DR-restricted peptides Fig. S13. Longitudinal assessment of SARS-CoV-2 T cell responses to HLA-DR- and HLA-A*24-restricted SARS-CoV-2-derived peptides Fig. S14. Gating strategy for flow cytometry-based evaluation of surface marker and intracellular cytokine staining Table S1. Clinical characteristics of convalescent donors Table S2. Characteristics of individual convalescent donors Table S3. PCR retesting of convalescent donors with post-infectious symptoms at T2 Table S4. SARS-CoV-2-specific and cross-reactive HLA class I- and HLA-DR-restricted T cell epitope compositions Table S5. Intensity of T cell and antibody response at T2 according to gender, age, and BMI Table S6. Longitudinal characterization of SARS-CoV-2-derived HLA-DR-restricted peptides Table S7. Longitudinal characterization of SARS-CoV-2-derived HLA-A*24-restricted peptides Table S8. Control peptides Data file S1. Raw data
